# A Wonderful Skull

**Published:** 1868-08

**Authors:** 


### ARTICLE VIII.

### *A Wonderful Skull.*

At a meeting of the Massachusetts Medical Society held recently, Dr. John M. Harlow, physician and surgeon, of Woburn, but formerly of Cavendish, Vermont, read a paper containing the history of a most interesting case of injury to the head, and presented to the meeting the veritable skull which sustained the injury.

This case occurred some twenty years ago, in Cavendish, Vt., and was described at length in the New Jersey *Medical Reporter* of that date—the predecessor of this journal.

On the 13th of September, 1848, Phineas P. Gage, foreman of a gang of men engaged in blasting a deep cut in the continuation of the Rutland and Burlington road, had a tamping iron blown through his brains, and recovered within sixty days, living twelve years after. The case caused great discussion when reported by Harlow in the medical journals at that time, and it was largely disbelieved, many eminent surgeons declaring the occurrence, as described, to be a physiological impossibility. Dr. Harlow, in presenting the paper, justly said, "that it is due to science that a case so grave, and succeeded by such remarkable results, should not be lost sight of; and that its subsequent story should have a permanent record.

Gage was a perfectly healthy, strong and active young man, 25 years of age, or nervo-bilious temperament, 5½ feet in height, average weight 150 pounds, possessing an iron will, as well as an iron frame; muscular system remarkably well developed, having had scarcely a days illness from childhood up.

As described in the paper read, it appears that a drilled hole had been charged with powder, and he was about tamping it in, (or, more popularly, raming it down,) when his attention was called for a moment. Looking over his shoulder at his men, he at the same moment rammed down the iron, supposing his assistant had poured sand on the powder, as is the custom. The iron struck fire from the rock, the charge exploded, and the iron was driven up into his cheek and out of the top of his head, high in the air, and was afterward found several rods distant, smeared with blood and brains.

The tamping iron was 3½ feet in length, 1¼ inches thick, and pointed at one end; the taper being seven inches long, and the diameter of the point a quarter of an inch. It weighed thirteen pounds. The point was upward, and the iron smooth.

The missile entered, by its pointed end, the left side of the face, immediately anterior to the angle of the lower jaw, and passing obliquely upward and slightly backward, emerged out of the top of the head in the median line, at the back part of the frontal bone, near the coronal suture. The ordinary reader will understand it better, if we say that, pointing upward, it entered the cheek outside the teeth, and under the cheek bone, went inside an inch behind the eye, and out of the top of the head in the centre, two inches back of the line where the forehead and hair meet.

The patient was thrown on his back, and gave a few convulsive motions of the extremities, but spoke in a few minutes. He was taken three-quarters of a mile in a sitting position in a cart; got out the cart himself with the aid of his men, and an hour afterward, with the assistance of Dr. Harlow holding his arm, walked up a flight of stairs to his room. He was conscious, but exhausted from loss of blood, which found its way from the mouth into the stomach, and was ejected as often as every fifteen or twenty minutes by vomiting. His bed and person were soon a gore of blood.

One piece of the skull had been broken out in fragments; another piece was raised and thrown back, like a door, the scalp serving as a hinge; and on the opposite side of the wound there was another fracture and an elevation. The globe of the left eye was partially protruded from its orbit, the left side of the face was more prominent than the right. The opening in the skull was two inches wide by three and a half long, and the brain was hanging in shreds on the hair. The pulsation of the brain could be distinctly seen, and the doctor passed his finger in its whole length without the patient saying he felt pain.

The paper gives an account of the treatment of the case. In fifty-nine days the patient was abroad. On the third day there were inflammation and some delirium; and during several weeks there was occasional delirium; for two weeks of the time the patient lay in a stupid condition, and his death was expected, and his grave-clothes prepared. On the 25th of November he went in a close carriage 30 miles to his home in Lebanon.

The subsequent history of the case is interesting. Gage came back to Cavendish in April, in fair health and strength, having his tamping iron with him, and he carried it with him till the day of his death, twelve years after. The effect of the injury appears to have been the destruction of the equilibrium between his intellectual faculties and the animal propensities. He was now capricious, fitful, irreverent, impatient of restraint, vacillating, a youth in intellectual capacity and manifestations, a man in physical system and passions. His physical recovery was complete, but those who once knew him as a shrewd, smart, energetic, persistent business man, recognized the change in his mental character. The balance of his mind was gone. He used to give his nephews and his nieces wonderful accounts of his hair-breadth escapes, without foundation in fact, and conceived a great fondness for pets.

He went to various places, being engaged here and there; was a year and a half in charge of horses at a livery stable; was exhibited at Barnum's Museum in New York; and in August, 1852, four years after his injury, left New England forever, and went to Valparaiso with a man who was going to establish a line of coaches. Here he lived eight years, occasionally driving a six-horse coach, and enduring many hardships. In 1859 his health began to fail; in 1860 he had a long illness, the nature of which cannot now be ascertained.

He now left Chili, and Dr. Harlow lost all trace of him for some years, but finally found out that the mother and sister were in San Francisco, wrote to them, and ascertained that Gage had got there in 1860; worked with a farmer at Santa Clara, and in February 1861, was taken with epileptic fits; afterward he worked in several places; and finally in May, 1861, had a succession of fits, which lasted a couple of days, and carried him off. There was no autopsy made, Dr. Harlow made overtures for the possession of the skull, on account of its scientific interest, and the world at large is under obligation to the relatives who were willing to surrender it for the uses of medical science. It appears that the man could see out of his left eye, though the lid was not fully subject to the will; that he was troubled with uneasiness in the head.

Dr. Harlow, in summing up his valuable but interesting paper, presented these views: 1st. The recovery is attributed solely to the vis vitæ, vis conservatrix, or, if some like it, vis medicatrix naturæ. 2d. This case has been cited as one of recovery; physically the recovery was nearly or quite completed for the four years immediately succeeding the injury, but ultimately the patient succombed to progressive disease of the brain. Mentally the recovery was only partial; there was no dementia; intellectual operations were perfect in kind, but not in degree or quantity. 3d. Though the case may seem improbable, yet the subject was the man for the case, as his will, physique, and capacity for endurance could scarcely be equalled; the missile was smooth and pointed, dilating and wedging off rather than lacerating the tissues; the bolt did little injury till it entered the base of the brain, and that opening served as a drain for the blood and matter, and other substances that might have caused death by compression; the part of the brain traversed was the strongest for the purpose.

Dr. Harlow had with him and exhibited the skull and the iron.

The piece of skull, which was thrown backward like a door, and was afterward replaced, had grown to the opposite edge by a new formation of bone plainly marked; the holes were large and well defined; and the whole appearance of the skull proved the truth of the account; which had also been verified by letters from some of the first men at Cavendish, Vt. It appears that, early in the history of the case, a number of fragments of bone came down into the mouth through the opening in the inside, and were voided.

A great deal of interest was manifested in the examination of these important contributions to surgical science, and Dr. Harlow was abundantly complimented for the persistence with which he had followed up the case for nearly twenty years.—*Medical and Surgical Reporter.*

---

